# Why and how genetic canalization evolves in gene regulatory networks

**DOI:** 10.1186/s12862-016-0801-2

**Published:** 2016-11-08

**Authors:** Estelle Rünneburger, Arnaud Le Rouzic

**Affiliations:** Laboratoire Évolution, Génomes, Comportement, Écologie, CNRS–IRD–Univ. Paris-Sud–Université Paris-Saclay, Gif-sur-Yvette, 91198 France

**Keywords:** Genetic architecture, Quantitative genetics, Individual-based simulations, Evolution of epistasis

## Abstract

**Background:**

Genetic canalization reflects the capacity of an organism’s phenotype to remain unchanged in spite of mutations. As selection on genetic canalization is weak and indirect, whether or not genetic canalization can reasonably evolve in complex genetic architectures is still an open question. In this paper, we use a quantitative model of gene regulatory network to describe the conditions in which substantial canalization is expected to emerge in a stable environment.

**Results:**

Through an individual-based simulation framework, we confirmed that most parameters associated with the network topology (complexity and size of the network) have less influence than mutational parameters (rate and size of mutations) on the evolution of genetic canalization. We also established that selecting for extreme phenotypic optima (nil or full gene expression) leads to much higher canalization levels than selecting for intermediate expression levels. Overall, constrained networks evolve less canalization than networks in which some genes could evolve freely (i.e. without direct stabilizing selection pressure on gene expression).

**Conclusions:**

Taken together, these results lead us to propose a two-fold mechanism involved in the evolution of genetic canalization in gene regulatory networks: the shrinkage of mutational target (useless genes are virtually removed from the network) and redundancy in gene regulation (so that some regulatory factors can be lost without affecting gene expression).

**Electronic supplementary material:**

The online version of this article (doi:10.1186/s12862-016-0801-2) contains supplementary material, which is available to authorized users.

## Background

Canalization reflects the capacity of an organism’s developmental process to maintain a constant phenotype in spite of perturbations. This concept was first introduced by Waddington [1], who noticed the striking robustness properties of the development in complex organisms. This seminal work inspired further conceptual progress, in particular about the distinction between robustness to environmental versus genetic perturbations [2]. While environmental canalization can be easily explained by natural selection, evolution toward a lower sensitivity to mutations (which will be called indifferently genetic canalization or genetic robustness in this paper) is less straightforward. As genetic canalization tends to decrease the adaptation potential (by preventing the appearance of new phenotypes), it also paradoxically enhances long term evolvability, by allowing the accumulation of cryptic genetic variation (i.e. mutations sheltered from natural selection as they do not affect the selected phenotypes, and which can be released afterwards and thus contribute to rapid adaptation) [3–5]. Genetic canalization is thus largely overlapping with other evolutionary properties of populations, as exemplified by studies based on population genetic models [6–9]. In particular, counter-intuitively, robustness and evolvability may not systematically be opposite properties of genetic systems [10, 11].

Integrating evolution of genetic variation into the Darwinian framework is thus of outstanding interest in evolutionary biology [12]. However, the complexity of this task is such that it has to rely on formal modelling of realistic genetic architectures involving genetic interactions (epistasis). Simple models of gene regulatory networks appear as good candidates for studying the evolution of epistatic systems. In particular, even small regulatory networks display a wide range of different interaction patterns [13]. The gene regulation model proposed by A. Wagner [14, 15] has been widely used to understand the evolution of canalization in regulatory networks [4, 5, 16, 17]. Based on a regulation network encoded as an interaction matrix, it implements a dynamical model of gene expression along the development, and facilitates the theoretical study of long-term evolution under various selection pressures.

Independent implementations of this model, most of the time in individual-based evolutionary simulations, have confirmed consistently that genetic canalization can evolve without selecting directly for robustness. For instance, the sensitivity to mutations can decrease (i.e. genetic canalization can increase) as a by-product of stabilizing selection on gene expression at the end of the development [15, 18]. Using a similar framework, Siegal & Bergman [16] suggested that selection on the stability of development also leads to higher canalization, even in the absence of stabilizing selection for a phenotypic optimum. Other studies based on the same theoretical background have subsequently highlighted the relationship between the evolution of genetic canalization and structural properties of the network (such as density of interactions [15, 16, 19, 20], topology [21–23], size of the network [16]) or between canalization and various evolutionary properties (such as selection [24] or reproduction regime [25–27]).

Most of theses studies confirm that robustness is expected to evolve from complex genetic architectures, such as gene regulatory networks. However, (i) there is little empirical evidence that genetic canalization can actually evolve in real populations (in particular in sexual species) [28], (ii) quantitative characters are far from being completely canalized, as evidenced by the prevalence of monogenic diseases or lethal mutations, and by the high level of genetic variance in most natural populations [29], and (iii) little is known about how canalization translates into observable properties from real genetic systems. In this paper, we aim to challenge theoretically the limits and the prediction power of the “canalizing gene networks” theory. First, we defined the conditions in which substantial genetic canalization is expected to appear (or not appear) through indirect selection (“why canalization evolves”). Second, we described the mechanisms involved in the evolution of such genetic canalization in the simulated gene networks (“how canalization evolves”). These results thus bring expectations and conceptual tools for a future empirical validation of the canalization theory.

## Methods

In order to address the conditions and mechanisms involved in the evolution of genetic canalization, we used a simulation framework derived from the Wagner model [14], modified in such a way that gene expression could be treated as quantitative values. This setting makes it possible to use traditional quantitative genetics tools, and to relate the results to the evolution of quantative measurements of gene expression.

### Genotype-to-phenotype map

Each individual is characterized by its genotype (the matrix *W*, representing the interactions among transcription factors within the regulatory network), from which its phenotype (expression levels for all genes in the network) is calculated.

More specifically, the *L*×*L* interaction matrix *W* describes the way the *L* genes interact, each *W*_*ij*_ representing the effect of gene *i* on the expression of gene *j* [15–17]. This effect can be nil (no interaction, *W*_*ij*_=0), activating (*W*_*ij*_>0) or repressing (*W*_*ij*_<0). Each line of the matrix stands for an allele, i.e. a serie of potential fixation sites for regulation factors in the gene promoter. In the default set of simulations, individuals are diploid, and their genotype is obtained by averaging both parental alleles. We also ran simulations with haploid individuals, which showed very similar results (Additional file 1: Figures S1 and S2).

**Fig. 1:**
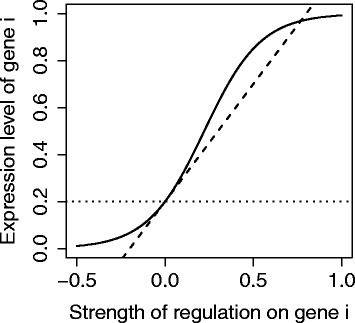
Expression level of gene *i* as a function of the strength of regulation: $S_{i_{t+1}}=f\left (\sum _{j} W_{ij} S_{jt}\right)$. The *dashed line* represents the slope *d* *f*/*d* *x*=1 at the point *f*(*x*)=0; the *dotted line* stands for the gene constitutive expression *a*=0.2

The *S*_*t*_ vector represents the expression level of the *L* genes of the network at developmental time *t*; gene expressions scale between 0 (no expression) and 1 (maximum expression). In absence of regulation, transcription leaks to a constitutive gene expression value *a*, set by default to *a*=0.2 (20 % of the max expression). For simplicity, at the beginning of the development, gene expression values are all set to *a*. Gene expressions then change during the 16 developmental time-steps of the individual, according to:

1 $$ S_{t+1} = f(W S_{t}),  $$

where *f*(*x*) is a sigmoid function (Fig. 1) such as:

2 $$  f(x) = \frac{1}{1 + \left(\frac{1}{a}-1\right) \exp\left(\frac{-x}{a(1-a)}\right)}.  $$

This function ensures the scaling of the *S*_*t*_ vector in (0;1), and has been parametrized so that the slope *d* *f*/*d* *x*=1 when *x*=0. Biologically, this sets the scale of the *W* matrix so that *W*_*ij*_ tends to the induced change in gene expression in absence of other source of regulation.

For each individual, we measured (i) the vector of mean gene expressions ($\overline {S}$) during the 4 last developmental time steps, and (ii) the vector of the variance of gene expressions (*V*_*S*_) during the same period. In this context, assuming that the development is long enough to reach a stationary state, *V*_*S*_ can be used as a measurement of developmental stability (*V*_*S*_>0 indicates a cyclic equilibrium state).

### Population genetics model

Individual-based simulations were run for *G* non-overlapping generations. Successive generations of constant population size *N* were produced by simulating sexual reproduction among hermaphrodite individuals. In the haploid model, selection was performed after gametogenesis, while selection took place after fertilization in the diploid model. For each offspring, two parents were drawn with probabilities proportional to their fitness (see below). Gametes were generated by picking randomly one allele in each parent, assuming free recombination between the *L* loci (i.e. between parental alleles, represented by matrix rows). There was no recombination between regulatory sites (within matrix rows).

Mutations occurred at rate *μ* per haploid genome, during gametogenesis. Mutations can affect one or several loci in an individual, but each locus can be mutated only once (with a probability *μ*/*L*). For each mutated locus, a random non-zero element on the matrix row is modified by adding a random Gaussian modifier centred around the former value and of standard deviation *σ*_*m*_.

Simulations were initialized with *N* genetically identical individuals (same *W* matrix). The complexity *c* of the network specifies the frequency of interactions (*W*_*ij*_≠0), these non-zero entries were set randomly. The strength of these interactions was initially drawn in a Gaussian distribution $\mathcal {N}(0.0,0.1)$, indicating a balance between weak enhancers and repressors at the beginning of the simulations. The mean $\overline {W}_{ij}$ at the end of the simulation was used as an indicator of the average direction of regulations, this measurement being analogous to the network excitation score defined in [30].

The fitness *ω*_*n*_ of an individual *n* (proportional to the probability of reproduction) depends on the proximity to the target gene expression (*d*_*n*_) and the gene expression stability during development (*k*_*n*_), such that *ω*_*n*_=*d*_*n*_×*k*_*n*_. The fitness component *d*_*n*_ follows a bell-shaped distribution

3 $$ d_{n} = \exp\left[{-s \sum\limits_{i=1}^{\ell} ({\overline{S}_{n_{i}}} - \theta_{i})^{2}}\right]  $$

where ${\overline {S}_{n_{i}}}$ is the mean expression of gene *i* in individual *n*, *θ*_*i*_ the phenotypic (expression) optimum for gene *i*, and *s* the strength of stabilizing selection. *ℓ*≤*L* represents here the number of genes in the network for which the expression has a direct impact on fitness.

In addition, genotypes leading to cyclic gene expression (unstable development) were affected by a fitness penalty. In practice, the fitness component associated with developmental unstability in individual *n* was

4 $$ k_{n} = \exp\left[{-s' {\sum\limits_{i}^{L}} V_{S_{n_{i}}}}\right],  $$

the selection coefficient being arbitrarily set to *s* ^′^=46,000, such that individuals with a substantial variance in gene expression at the end of their development *V*_*S*_>10^−4^ are unlikely to reproduce (*k*_*n*_<0.01).

### Implementation and parameterization

The core program was written in C++, the simulation pipeline in Bash and the statistical analysis in R. Simulations were run with the default parameters presented in Table 1 unless stated otherwise. Simulations in which at least one gene was disconnected from the network (no regulators) were discarded from the analysis. Results were averaged over 200 replicates for each parameter set, assuring that the graphical representations are not affected by sampling error.

**Table 1:** Default parameters of the model

	Parameter	Value
Number of generations	*G*	10,000
Population size	*N*	5,000
Number of loci	*L*	6
Number of loci under selection	*ℓ*	2
Initialization of the alleles		$\mathcal {N}(0.0,0.1)$
Mutation rate per haplotype	*μ*	0.01
Mutation effect	*σ*_*m*_	0.5
Matrix complexity	*c*	0.5
Constitutive expression	*a*	0.2
Strength of stabilizing selection	*s*	10
Phenotypic optimum	*θ*	0.5

For each gene *i* in the network, simulations provided the mean and the variance of mean gene expressions $\overline {S}_{i}$ in the population every generation. In addition, we estimated genetic canalization in the following way. Each gene *i* in individual *n* was featured by a canalization score $C_{n_{i}} = -\log \text {Var}(M_{n_{i}})$, where $\text {Var}(M_{n_{i}})$ was the variance in the expression of gene *i* among mutants that differ from individual *n* by a single haploid mutation (drawn in a Gaussian distribution of mean 0 and s.d. 0.5) anywhere in the network. As a consequence, individuals displaying high robustness to mutations (small $\text {Var}(M_{n_{i}})$) were featured with high canalization scores. In practice, the genotype of each individual *n* in the population was mutated 100 times, and the average canalization in the population $\overline {C}_{i}$ was obtained by averaging out all $C_{n_{i}}$. An alternative measurement $C^{\prime }_{n_{i}} = \log \sqrt {\text {Var}(M_{n_{i}})}/S_{n_{i}}$, analogous to a coefficient of variation scaled by the phenotypic expression, gives essentially the same results (Additional file 2: Figure S3).

In addition, the mechanisms underlying the evolution of canalization were investigated through two specific indices, *R*_*s*_ and *D*_*s*_, that were calculated on every gene of the network. $R_{s_{i}}$ is the redundancy score of gene *i*, i.e. the average effect of knocking down another gene of the network on the expression of *i*. $D_{s_{i}}$ is the indispensability score for gene *i* and quantifies the average effect of knocking down this gene (gene expression forced to zero) on the selected genes (average absolute value of gene expression differences). Hence, a gene with a low *R*_*s*_ tends to be regulated in a redundant way (knocking down a single regulator is not enough to affect its expression substantially), while a gene with a low *D*_*s*_ is dispensable (its extinction does not influence the rest of the network).

## Results

### Evolution of gene expression and genetic canalization

We first studied how the simulated populations responded to selection and how canalization evolved. In the default parameter set (Table 1), two genes (out of six) were under selection pressure toward an intermediate optimal expression of *θ*=0.5, while the others could evolve freely. In parallel, we ran equivalent control simulations without any selection on gene expression. In these control cases, gene expression could thus drift freely, but gene networks remained constrained by selection against developmental instability.

**Fig. 2:**
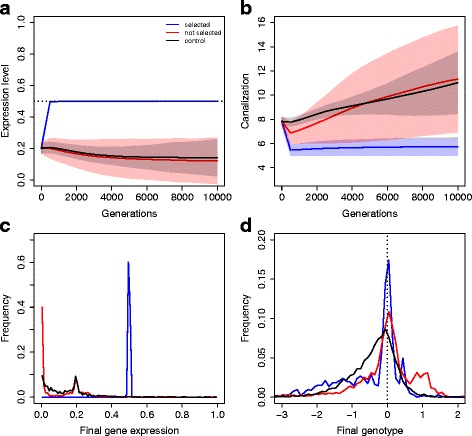
Evolution of canalization. Genes under direct and indirect selection pressures evolved differently. **a** Evolution of gene expression. *Blue*: selected genes (two out of six genes in the network); *red*: unselected genes; *black*: control simulations (no selected genes). The horizontal dotted line stands for the selected expression level. Filled areas stand for the s.d. across 200 replicates, illustrating the amount of stochasticity in the simulations. Statistics were measured every 500 generations. **b** Evolution of canalization score *C*_*i*_ (negative log of the variance of mutational effects). **c** Distribution of gene expression values *S*_*i*_ at the last generation (*G*=10,000). **d** Distribution of regulatory effects (*W*_*ij*_) at the last generation (immutable 0s are discarded from the analysis). The vertical dotted line stands for the absence of regulation

Unsurprisingly, genes under stabilizing selection consistently reached a value close to their optimal expression (Fig. 2 a and c). Such selection response was fast (less than a few hundred generations) and was associated with a substantial decanalization of the network (Fig. 2 b), which is an expected consequence of directional selection. Two comparisons are meaningful. Comparing selected (blue line) and unselected (red line) genes of a same network higlights patterns only due to the direct effect of stabilizing selection. Comparing unselected gene (red lines) and control simulation (black line) underlines the effects of indirect stabilizing selection on correlated genes. Freely-evolving genes, although epistatically associated with selected genes, displayed a gene expression pattern close to the control case.

However, this similarity between unselected and control case genes vanished when other network properties were considered, as the distribution of regulatory genotypes (*W*_*ij*_ values) was different both in mean and variance (Fig. 2 d). Control gene networks tended to evolve (on average) negative *W* ($\overline {W}_{ij}=-0.40\, \pm \, \text {s.e.}=0.01$), indicating a repressive matrix due to the strong selection against developmental instability. This repressive matrix was also observed for selected genes ($\overline {W}_{ij}\,=\,-0.50 \,\pm \, 0.03$), but was almost absent for unselected genes ($\overline {W}_{ij}=-0.03 \pm 0.02$). Moreover, both selected and unselected genes showed a peak around 0, corresponding to a strong signal for selected loss of interaction.

Further, the effect of mutations in the network on selected genes remained constant through time, while unselected genes evolved toward canalization (Fig. 2 b). After 10,000 generations, selected genes were relatively decanalized, as a canalization score of *C*∼5 corresponds to a large phenotypic effect of mutations (s.d.=0.08 for a trait ranging from 0 to 1). In contrast, unselected genes were substantially canalized (*C*∼11 is equivalent to a phenotypic s.d.=0.004). These results mean that a random mutation anywhere in the network has on average less effect after several thousand generations, this phenomenon being largely due to the canalization of unselected genes.

### Conditions for evolving canalization

**Fig. 3:**
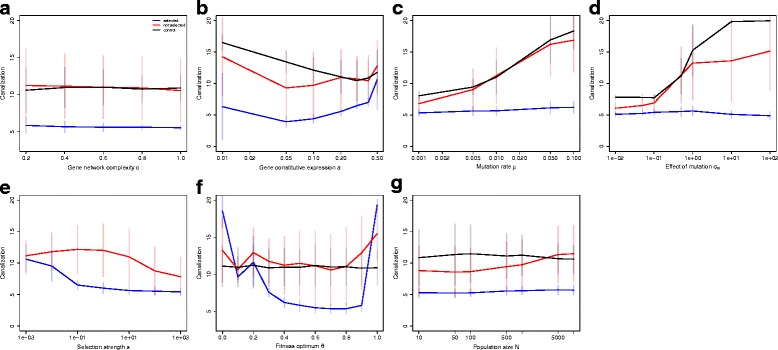
Why canalization evolves. Average and s.d. canalization scores (negative log of the variance of mutational effects) at *G*=10,000 generations; two genes out of six are under direct selection pressure. **a** Network complexity *c*. **b** Constitutive gene expression *a*. **c** Mutation rate *μ*. **d** Effect of a mutation *σ*_*m*_. **e** Strength of stabilizing selection *s*. **f** Fitness optimum *θ*. **g** Population size *N*

We then tested the influence of each parameter on the evolution of canalization (Fig. 3). Changing the complexity *c* of the network (Fig. 3 a) had limited effects on the evolution of canalization. Varying the gene constitutive expression *a* (Fig. 3 b) showed that the canalization score is higher when the constitutive expression is close to the phenotypic optimum *θ*=0.5 (in other words, evolving canalization is easier close to the constitutive expression). The overlap of non-selected genes and control simulations is not a general phenomenon, as lower *a* makes evolution of canalization more difficult in selected networks, while larger *a* makes canalization easier. The largest levels of canalization in selected networks could be found for symmetric sigmoid functions (*a*=0.5), which is a common (but somehow unrealistic) setting in similar models.

In contrast, inflating mutational parameters (mutation rate *μ* and mutation size *σ*_*m*_, Fig. 3 c and d) increased the network robustness to mutations by orders of magnitude (from *C*∼7 to *C*∼15 on a logarithmic scale), equivalent to a drop in mutational standard deviation by a factor 60 (from s.d. (*M*_*n*_)=0.03 to s.d. (*M*_*n*_)=0.0005). This evolution of canalization concerned to a large extent unselected genes only. Overall, the effect of the different parameters on genetic canalization are scaled down for smaller mutation rates, but the main patterns remain very similar (Additional file 3: Figure S4). This is consistent with the theoretical prediction that selection for mutational robustness depends directly on the size and frequency of deleterious mutations. Selection to decrease mutation size remains a weak secondary selection strength, which is illustrated by the fact that it was less efficient in small populations (Fig. 3 g).

Selection for developmental stability (control simulations) featured high levels of canalization. This is consistent with the unconstrained evolution of these networks toward a low-expression, developmentally-stable state (Fig. 2 c). Paradoxically, the average canalization score decreased notably when adding stabilizing selection toward a phenotypic optimum (Fig. 3 e). This indicates that the production of a stable optimal gene expression was possible (i.e. the population lies close to the fitness optimum), but this precludes the evolution of canalization.

### Intermediate versus extreme gene expression

Strikingly, the value of the selected optimum had a huge effect on genetic canalization (Fig. 3 f). Extreme expression levels (i.e. no expression or maximal expression) could be extremely canalized, while intermediate expression values were unable to become mutationally robust.

**Fig. 4:**
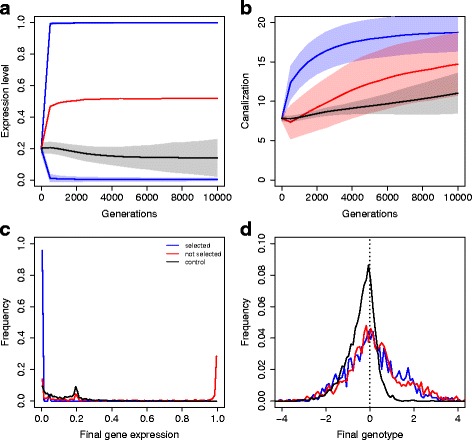
Extreme phenotypic optima. In a six-gene network, one gene is selected for the minimum expression, one for the maximum expression, the four remaining being free to evolve. **a** Evolution of gene expression. Filled areas stand for the s.d. across 200 replicates (not shown for the non-selected genes as their distribution is bimodal). Statistics were mesured every 500 generations. **b** Evolution of canalization score (negative log of the variance of mutational effects). **c** Distribution of gene expression values at the last generation (*G*=10,000). **d** Distribution of regulatory effects (*W*_*ij*_) at the last generation (immutable 0s are discarded from the analysis). The vertical dotted line stands for the absence of regulation

To better explore this difference between extreme or intermediate expression level, we ran additional simulations in which one gene out of six was selected toward 0, the second toward 1, and the others four remained free of direct selection pressure, mimicking on/off selection pressures (Fig. 4). In general, the average expression level in such networks was larger, and about 50 % of unselected genes were highly expressed at the end of the simulation (Fig. 4 c). Both selected and unselected genes were canalized very efficiently (faster than in control simulations, Fig. 4 b). Average regulatory effects were also larger than in control simulations, and were clearly positive in both cases ($\overline {W}_{ij}=0.27 \pm 0.07$ and $\overline {W}_{ij}=0.22 \pm 0.03$ for selected and unselected genes respectively, $\overline {W}_{ij}=-0.40 \pm 0.01$ for the control simulation, Fig. 4 d).

**Fig. 5:**
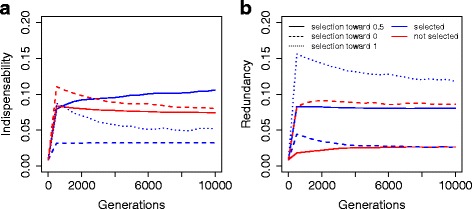
Redundancy and indispensability *Blue*: selected genes (two out of six genes in the network); *red*: unselected genes; *solid lines*: simulation from Fig. 2, with genes selected toward intermediate optima; *dashed and solid lines:* simulation from Fig. 4, with genes selected for extreme optima, respectively 0 and 1. **a** Redundancy index. Low values indicate a gene regulated in a redundant way. **b** Indispensability index. Low values indicate a dispensable gene

This phenomenon can be explained by the saturation of regulatory effects in the vicinity of extreme expression levels. Indeed, genes achieved full expression or repression through the unlimited accumulation of multiple regulatory factors. This was confirmed by the analysis of redundancy scores (Fig. 5 a). The figure illustrates how selected genes tend to become more robust to knock-downs of regulatory factors when selected for extreme optima, while genes selected for intermediate expression are unable to change their redundancy level.

### Degrees of freedom in the network

**Fig. 6:**
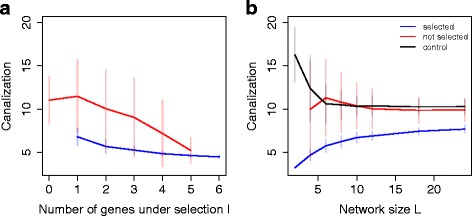
Degrees of freedom. **a** Number of genes under selection in a six-gene network. **b** Network size (two genes under selection)

In the default simulations, only two genes out of six were submitted to direct selection pressure, allowing four degrees of freedom in the network (i.e. expression levels of these four genes could change due to drift and/or indirect selection without affecting fitness). We explored the effect of global selection constraints on the evolution of canalization, keeping the number of genes unchanged and applying stabilizing selection on a subset of genes ranging from one to all six genes (Fig. 6 a). We observed that networks with more degrees of freedom tended to evolve more canalization than completely constrained networks. More specifically, both directly selected and unselected genes were more canalized in networks with only one target gene. Interestingly, from two to six genes under selection, canalization could not evolve for target genes, but the rest of the network could still benefit from additional degrees of freedom.

We also ran simulations in which the number of genes under selection did not change (two selected genes), but the global number of genes in the networks varied. The proportion of selected genes thus changed accordingly, while keeping constant the global selection pressure (Fig. 6 b). Adding degrees of freedom in such a way enhanced the canalization score, and which was not only due to an increase in network size (control simulations showed decreasing canalization). Interestingly, robustness stabilized for unselected genes above 10 genes, but kept increasing for genes under selection.

The analysis of the indispensability scores (i.e. the influence of each gene on the expression of selected genes) illustrates how unselected genes lose their influence on the rest of the network during evolution, parallel to the increase in genetic canalization (Fig. 5 b).

## Discussion

### Model approximations

Although our approach remains theoretical and cannot accurately represent all details of molecular and developmental mechanisms, gene network-based models derived from Wagner [15] have been widely used to study the evolutionary properties of complex genetic architectures. In an attempt to get more insights into the evolutionary changes associated with genetic canalization, we slightly modified this traditional framework toward more realistic assumptions, namely: (i) as in recent modeling work [19], gene expressions are quantitative variables ranging from 0 (no expression) and 1 (maximum expression); and (ii) we used an asymmetric regulation sigmoid function, featuring a 20 % constitutive gene expression simulating transcription leak. This contrasts with most gene network models, which generally consider that unregulated genes are either completely silent or substantially expressed, none of these alternatives appearing as satisfactory when focusing on the strength of selection on regulatory sites. The shape of the sigmoid normalizing function is of importance, as its slope conditions the genotype-phenotype mapping, and explains the change in the effect of mutations as a function of gene expression (intermediate expressions being more difficult to canalize than than extreme expressions [31]).

In our simulation study, we had to deal with computational constraints, limiting the population size, the number of genes, and the number of simulated generations. However, we could show that population size has only a minor influence on the final results, and we are thus confident that our results obtained with *N*=5,000 individuals can be extrapolated to more realistic conditions. Consistently with the literature, network size quantitatively affects the evolution of canalization, larger networks being more robust [15, 16]. This effect, however, remains moderate compared to mutational parameters. In order to speed up simulations so that 10,000 generations are enough to get insights into long-term evolutionary properties, we used the common trick of increasing the mutation rate close to the largest existing estimates (*μ*=0.01 per gamete and per generation). Simulations with smaller mutation rates (Additional file 3: Figure S4) confirmed the qualitative validity of the large mutation rate simulation strategy.

### Why canalization evolves

In this paper, our aim was to identify the conditions in which genetic canalization can evolve in gene networks, as well as the parameters influencing it. In particular, we confirmed that the evolution of canalization was mainly influenced by mutational parameters, canalization being maximal for high mutation rates and high mutation sizes. This result is consistent with previous studies [32, 33]. The impact of mutations on gene network evolution is two-fold: (i) mutations allow the population to spread over the neutral network space and to explore it faster [11], and (ii) a higher frequency of deleterious mutants increases the strength of indirect selection in favor of mutationally-robust genotypes [34].

We also established that the second parameter of great influence on the level of canalization in a network is the gene expression level. Much more genetic robustness occurs when natural selection favors extreme gene expression values (all or nothing) rather than intermediate ones. Many previous studies generally considered selection toward extreme optima, thus focusing on a particular case that is the most favorable to the evolution of canalization. This new result thus highlights that the theoretical literature probably overestimates the generality of genetic canalization.

Most parameters influencing the gene network structure and topology (number of genes, network complexity) seem to have only moderate effects on canalization (see e.g. [21, 30] for a similar result). Hence, the propensity for a given network to be canalized can hardly be deduced from its basic topological features.

As a consequence, our results suggest that gene networks in various organisms may realistically harbour different canalization levels. The most canalized networks are expected to be found in genetic systems involving large population sizes, high mutation rates, and low selection levels (both in terms of selection strength and number of genes that influence directly the selected phenotypes). Larger gene networks are also expected to evolve (slightly) larger levels of canalization. In addition to these classical population genetic parameters, our results evidence a direct impact of the selected expression level on genetic canalization.

### How canalization evolves

**Fig. 7:**
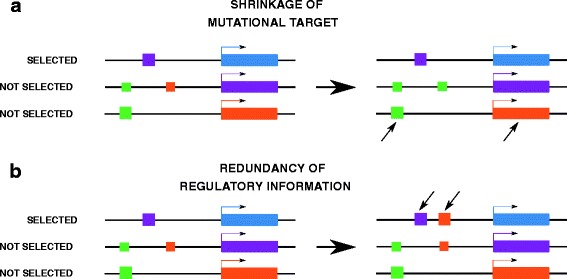
How canalization evolves. We identified two main processes involved in the evolution of canalization: **a** shrinkage of mutational target and **b** redundancy of regulatory information. The blue gene is the output of the network (the only one having an effect on fitness). *Square* boxes represent regulatory sites (in this example, all interactions are enhancing gene expression), *rectangles* represent genes. The box colors match the color of the corresponding regulatory gene, and their size is proportional to the effect of the regulation (two small boxes have the same effect as one big box). The green gene (not shown) is assumed to be expressed constitutively. *Black arrows* indicate locations (either regulatory sites or gene sequences) which will be canalized (mutations in these sequences have no effect on fitness). In **a**, the replacement of a weak orange site by a weak green site excludes the orange gene from the “useful” network. In **b** the new strong orange regulator is redundant and can compensate the loss of both sensitivity or expression of the purple gene

It is usually considered that regulatory gene networks are typical instances of complex systems, whose emergent properties cannot be understood by reductionist approaches based on single genes. Genetic or environmental canalization are often described as examples of such emergent properties. Unfortunately, this reasoning tends to consider genetic systems as undecipherable black boxes described by their properties rather than their internal mechanisms. This makes it extremely complicated to deduce evolutionary properties of a system from basic genetic knowledge (function and biochemical properties of genes and proteins). Here, we identified two mechanisms prone to explain the general canalization features of gene regulatory networks without involving emergent properties *stricto-sensu*: (i) shrinkage of mutational target, and (ii) redundancy of regulatory information (Fig. 7).

First, when some genes are not under the direct influence of natural selection, evolution in stable environments tends to favour genotypes in which such non-selected genes can be altered without influencing the important output of the network. This phenomenon is equivalent to a reduction of the network size, as some genes become virtually disconnected from the rest of the network. As a consequence, the number of mutations affecting the ‘useful’ genes in the network tends to decrease, and this shrinkage of mutational target leads to a progressive reduction in the mutational variance along generations. Interestingly, the directly-selected genes remain affected by *cis* mutations, but the lesser amount of epistatic interactions decreases the probability of being affected by *trans* mutations.

The second mechanism relies on the accumulation of redundant regulatory information, in such a way that the loss of one of the regulatory factors does not alter the expression of the target genes. In our model, this can happen only for target genes selected for extreme expression levels, due to a saturation effect linked to the shape of the sigmoid regulation-expression function (Eq. 2). This mechanism explains why canalization can evolve so easily for extreme optimum selection levels, which is the usual fitness landscape in Wagner-like models, even those based on sigmoid regulation expression functions [16, 21, 25, 35]. These two mechanisms are non-exclusive, i.e. mutational target shrinkage is generally associated with redundancy. “Useless” genes become innocuous by evolving low interactions with selected genes and accumulating redundant repressors that canalize them into a developmentally stable, low-expression state. Whether or not these “simple” mechanisms are sufficient to explain the evolution of canalization in gene networks remains an open question. As a matter of fact, in our simulations, we could hardly observe evolution toward canalization in conditions where both of these mechanisms were hampered (direct selection on all genes, which prevents mutational target shrinkage, and selection for intermediate expression values, which prevents redundancy). This observation does not necessarily mean that more subtle emergent properties cannot evolve from complex gene networks. In particular, we have not modeled any complex selection pressure (e.g. favoring phenotypic plasticity [4, 15] or fluctuating selection targets [30, 36]).

## Conclusion

The knowledge on how natural selection affects gene regulatory networks is so tenuous that inferring realistic selection conditions for the evolution of canalization in real gene networks has to rely on speculations. On one hand, it seems unlikely that all transcription factors are systematically targeted by direct selection. Most regulatory genes are only known to affect other transcription factors, and most networks seem to have a substantial amount of degrees of freedom (more network genes than independent output). On the other hand, assuming that natural selection favours systematically extreme gene expression levels (no expression or maximal expression) is clearly more problematic. For instance, most transcriptomic studies focus on gene expression differences between genotypes or environmental conditions, bringing convincing evidence that quantitative (and sometimes modest) expression variations may have major impacts on physiology and development. It would be indeed surprising that selection optima on complex physiological processes are not intermediate. Our results thus suggest that gene networks underlying such subtle regulatory processes may be less prone to evolving strong long-term genetic canalization than previously thought.

## Additional files

Additional file 1
**Figures S2 and S1.** Ploidy level. **Figure S2.** Effect of ploidy on the evolution of expression and canalization. Solid lines stand for diploid populations, dashed lines for haploid populations, and dotted lines for haploid populations with $\sigma _{m}^{\prime } = 2^{1/2} \sigma _{m}$ for the canalization tests (to compensate the increase in mutational effect that comes from the second unchanged haplotype in diploids). Subfigure indexes match figure numbers from the main text. (2a)(2b) Evolution of gene expression and canalization in networks in which genes are selected towards an intermediate optimum. (4a)(4b) Evolution of gene expression and canalization in networks in which genes are selected towards extreme expression. **Figure S3.** Effect of simulation parameters on canalization in haploid *vs*. diploid populations. Average and s.d. canalization scores at *G*=10,000 generations; two genes out of six are under direct selection pressure. Solid lines stand for diploid populations, dashed lines for haploid populations. Subfigure indexes match figure numbers from the main text. (3a) Network complexity *c*. (3b) Constitutive gene expression *a*. (3c) Mutation rate *μ*. (3d) Effect of a mutation *σ*_*m*_. (3e) Strength of stabilizing selection *s*. (3f) Fitness optimum *θ*. (5b) Population size *N*. (5b) Network size *L*. (ZIP 14 kb)

Additional file 2
**Figure S3.** Scale effect on canalization scores. **Figure S4.** Canalization measured as coefficient of variation. Evolution of canalization when measured relative to the phenotypic expression (i.e. in a similar way as a coefficient of variation: $C^{\prime }_{n_{i}} = \log \sqrt {	ext {Var}(M_{n_{i}})}/S_{n_{i}}$. In practice, we computed $\overline C^{\prime }_{n} \simeq \frac{1}{2}\overline {C}_{n} - \log \overline {S}_{n}$. Subfigure indexes match figure numbers from the main text, the *Y* axis represents $\overline C^{\prime }$ averaged over different categories of genes (selected, non-selected, and for subfigure (4b), selected for optima of 0 (top blue line) and 1 (bottom blue line)). (PDF 6 kb)

Additional file 3 Effect of mutation rate. **Figure S4.** Canalization still evolves at low mutation rates. Average canalization scores at *G*=10,000 generations and *μ*=0.0001 ; two genes out of six are under direct selection pressure. Subfigure indexes match figure numbers from the main text. (3a) Network complexity *c*. (3b) Constitutive gene expression *a*. (3e) Strength of stabilizing selection *s*. (3f) Fitness optimum *θ*. (3g) Population size *N*. (5b) Network size *L*. (PDF 6 kb)
