# Supplementary figures and images for: Why and how genetic canalization evolves in gene regulatory networks
Source: BMC Evol Biol. 2016 Nov 8;16:239. doi: 10.1186/s12862-016-0801-2 (PMC5100197; doi:10.1186/s12862-016-0801-2)

(2a)

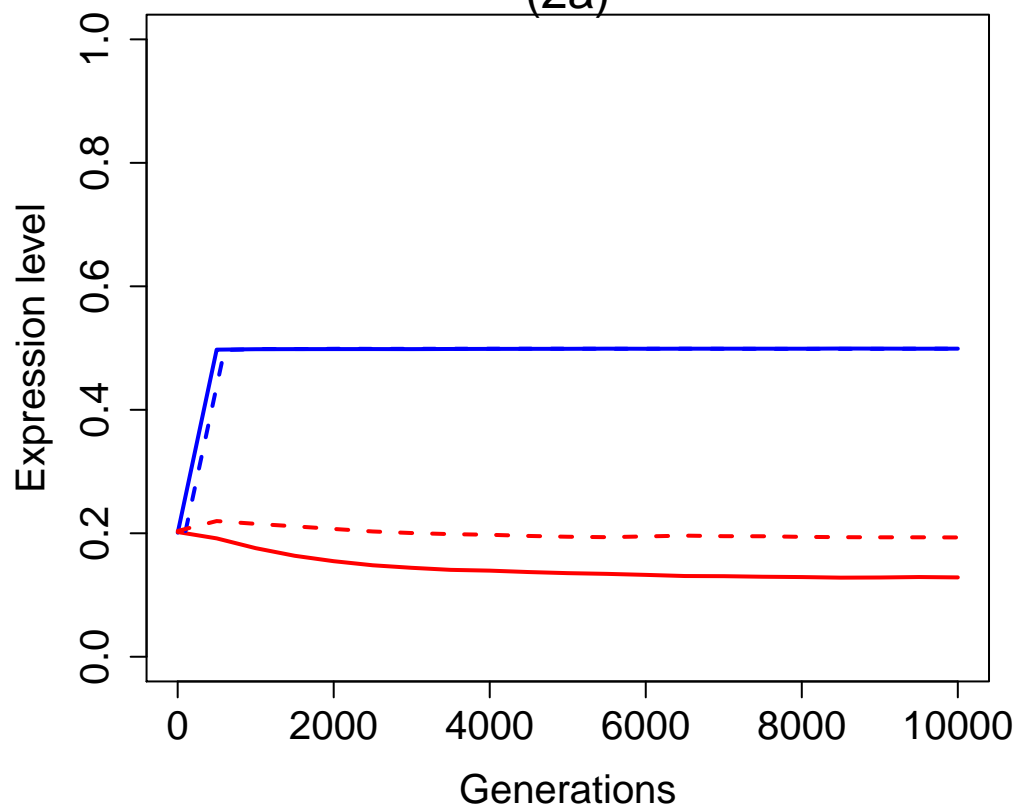

(2b)

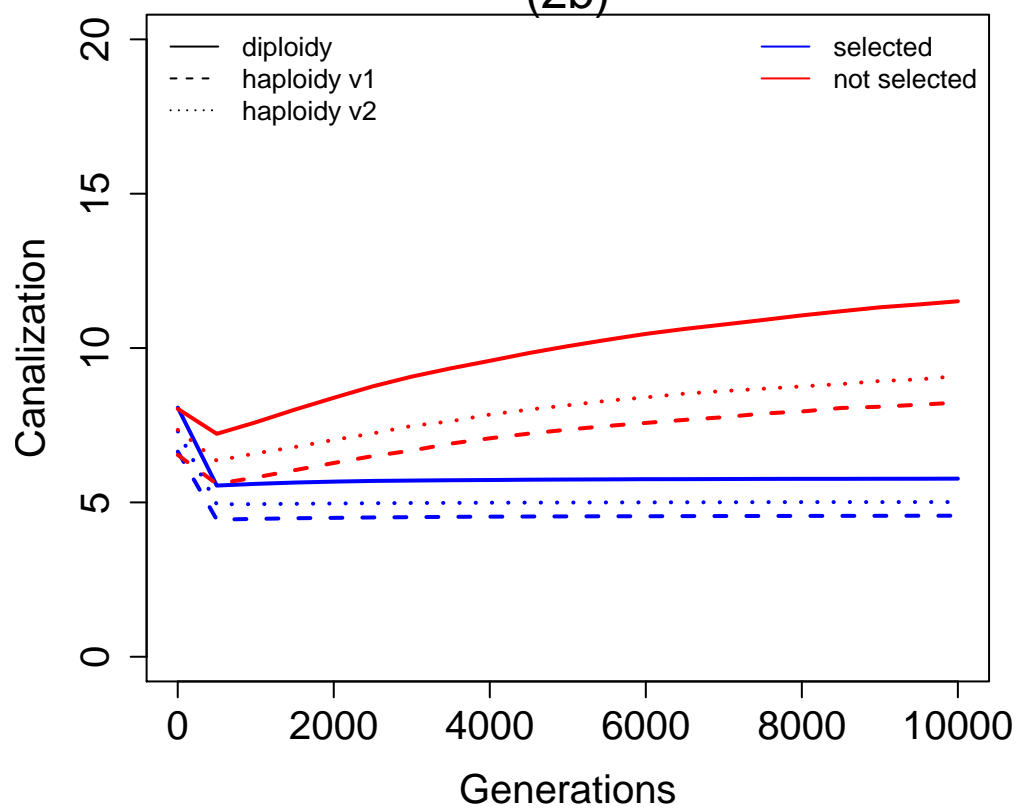

(4a)

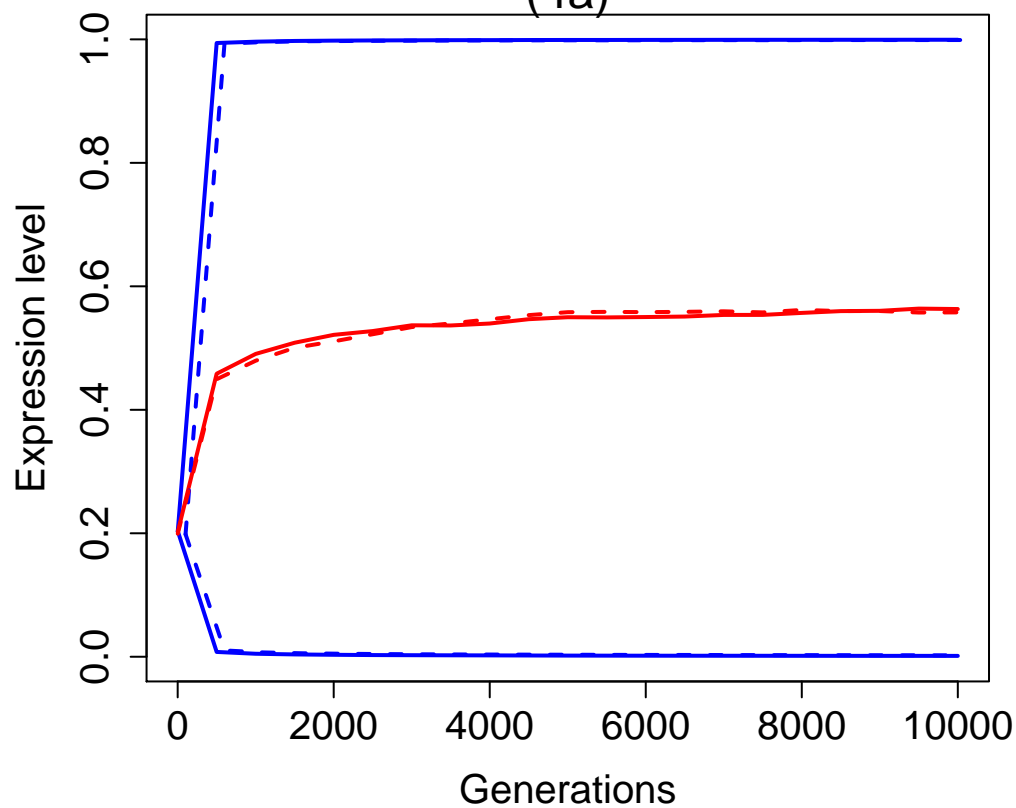

(4b)

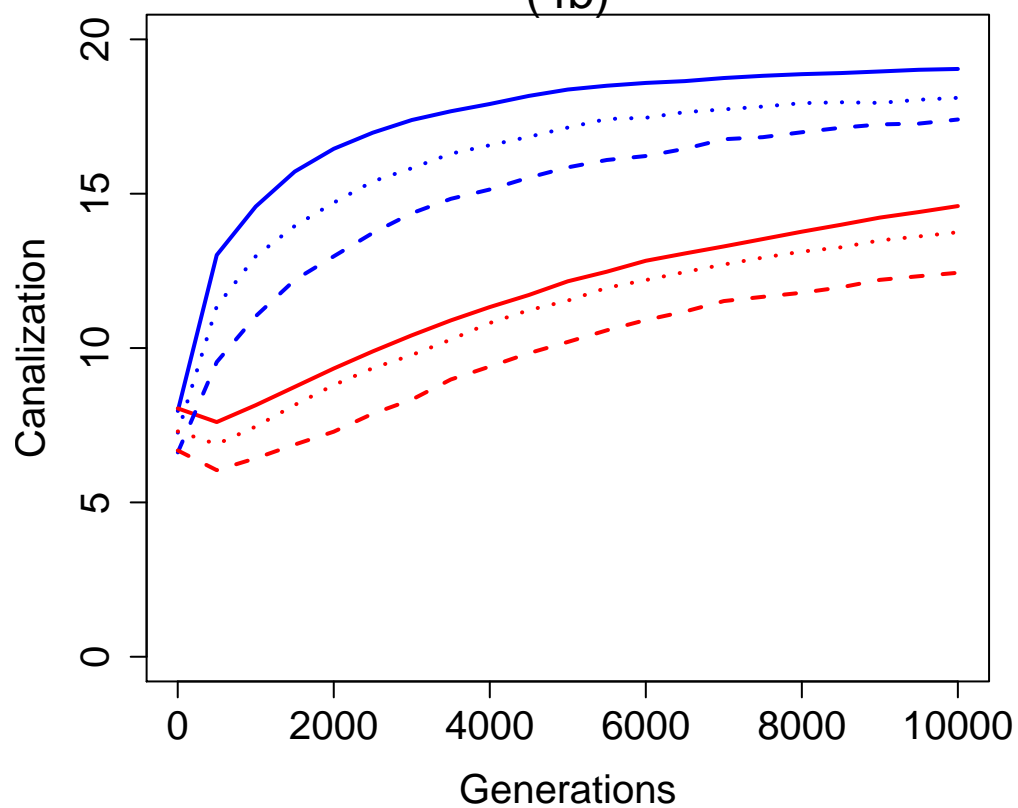

Supplement: Additional file 1 — Figures S2 and S1. Ploidy level. Figure S2. Effect of ploidy on the evolution of expression and canalization. Solid lines stand for diploid populations, dashed lines for haploid populations, and dotted lines for haploid populations with $\sigma _{m}^{\prime } = 2^{1/2} \sigma _{m}$σm′=21/2σm for the canalization tests (to compensate the increase in mutational effect that comes from the second unchanged haplotype in diploids). Subfigure indexes match figure numbers from the main text. (2a)(2b) Evolution of gene expression and canalization in networks in which genes are selected towards an intermediate optimum. (4a)(4b) Evolution of gene expression and canalization in networks in which genes are selected towards extreme expression. Figure S3. Effect of simulation parameters on canalization in haploid vs. diploid populations. Average and s.d. canalization scores at G=10,000 generations; two genes out of six are under direct selection pressure. Solid lines stand for diploid populations, dashed lines for haploid populations. Subfigure indexes match figure numbers from the main text. (3a) Network complexity c. (3b) Constitutive gene expression a. (3c) Mutation rate μ. (3d) Effect of a mutation σ m. (3e) Strength of stabilizing selection s. (3f) Fitness optimum θ. (5b) Population size N. (5b) Network size L. (ZIP 14 kb) [file 12862_2016_801_MOESM1_ESM.zip › supplementary_figure_2.pdf]

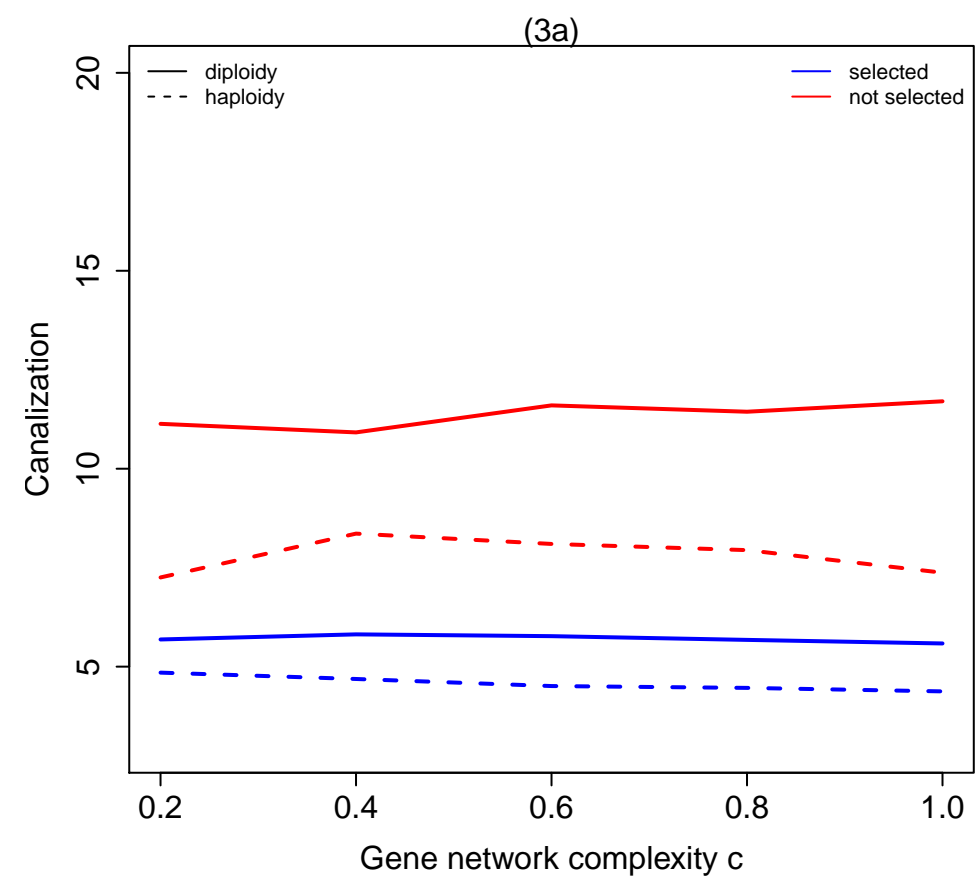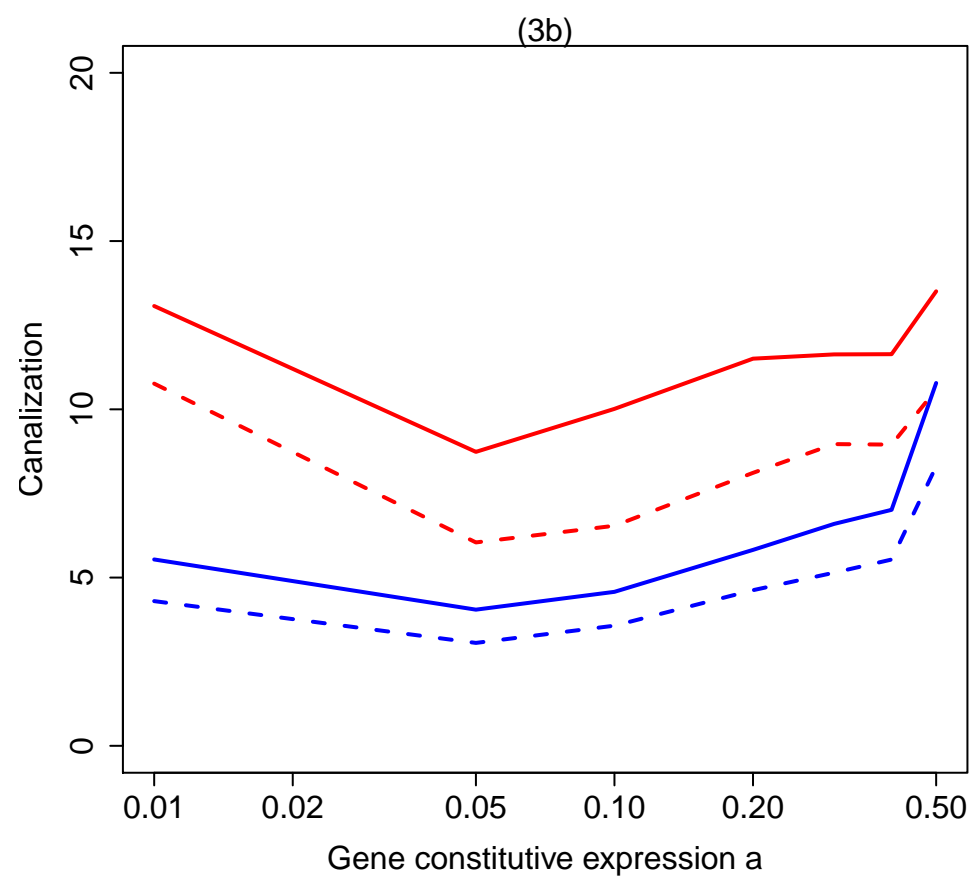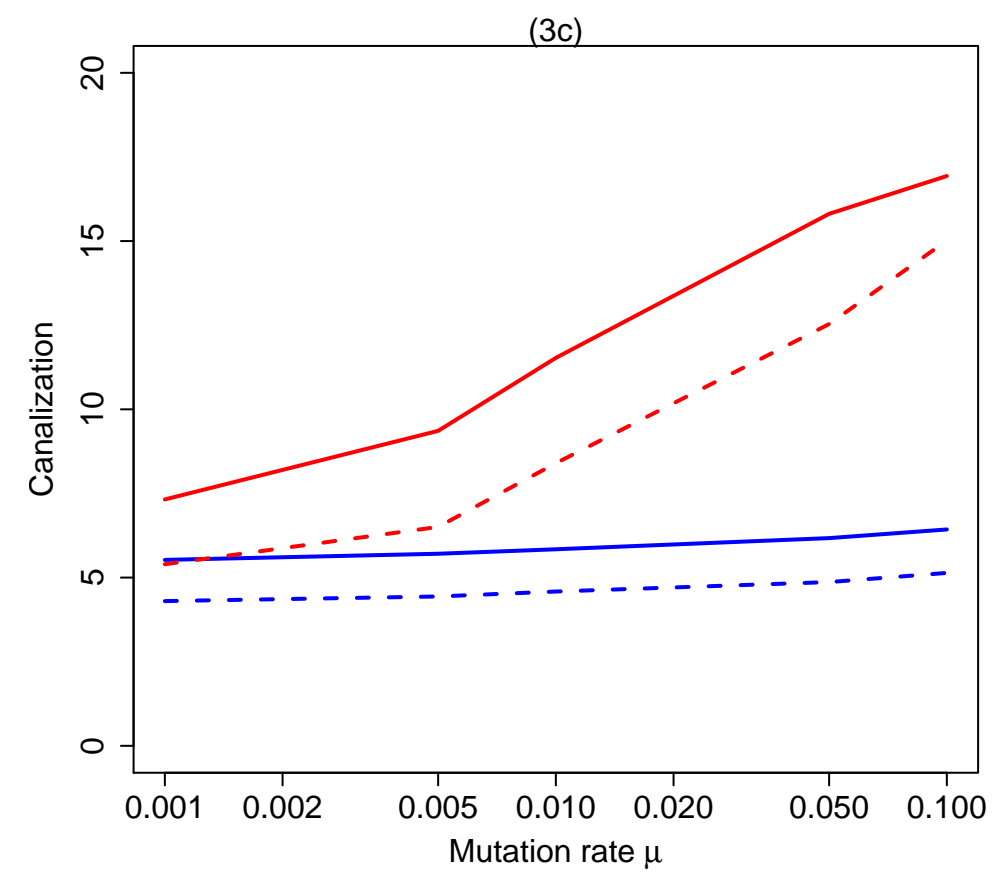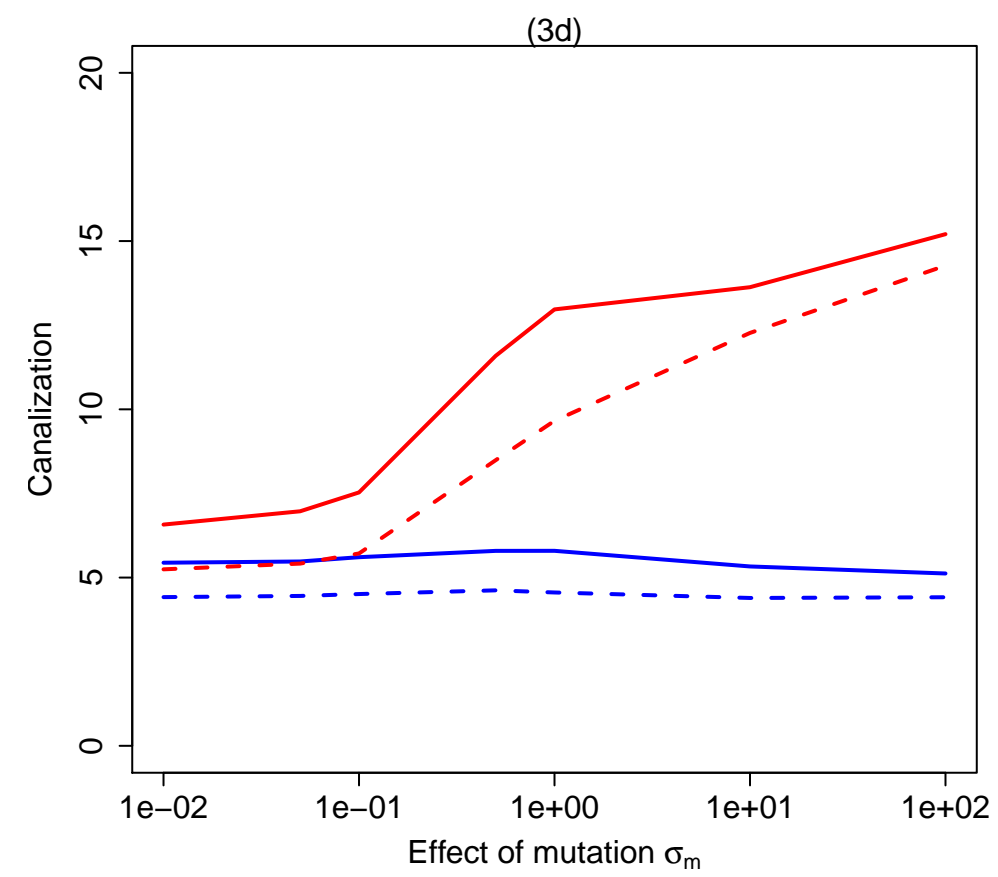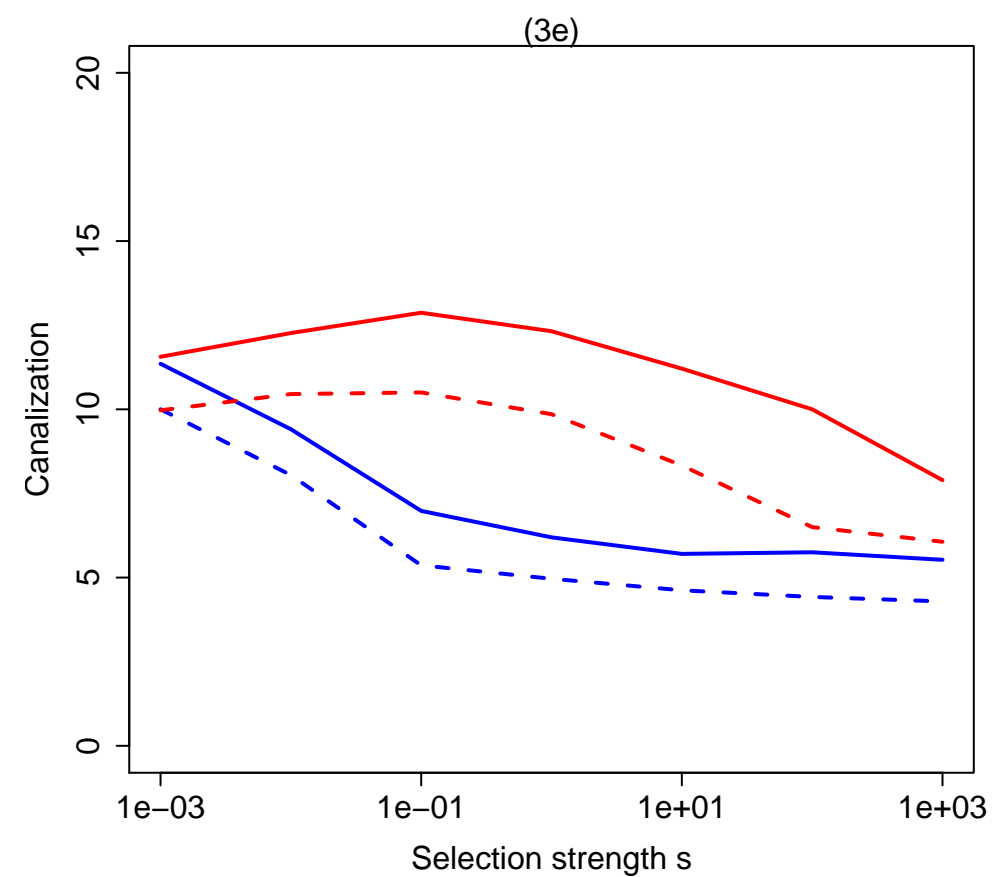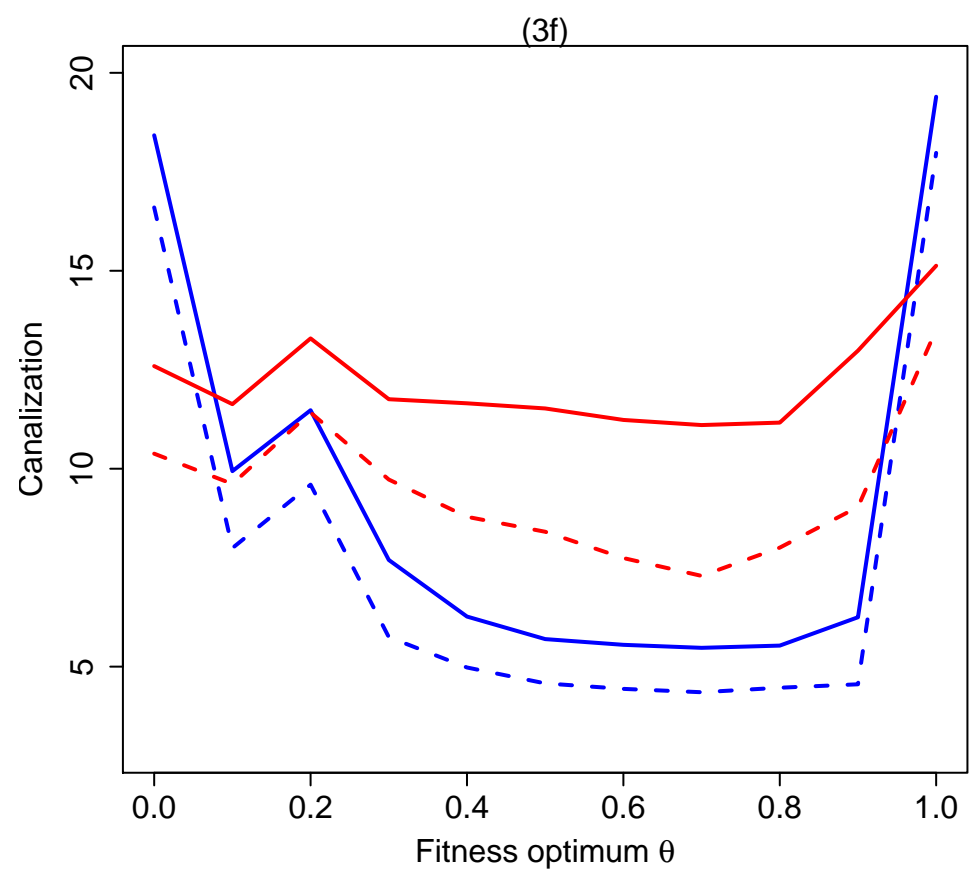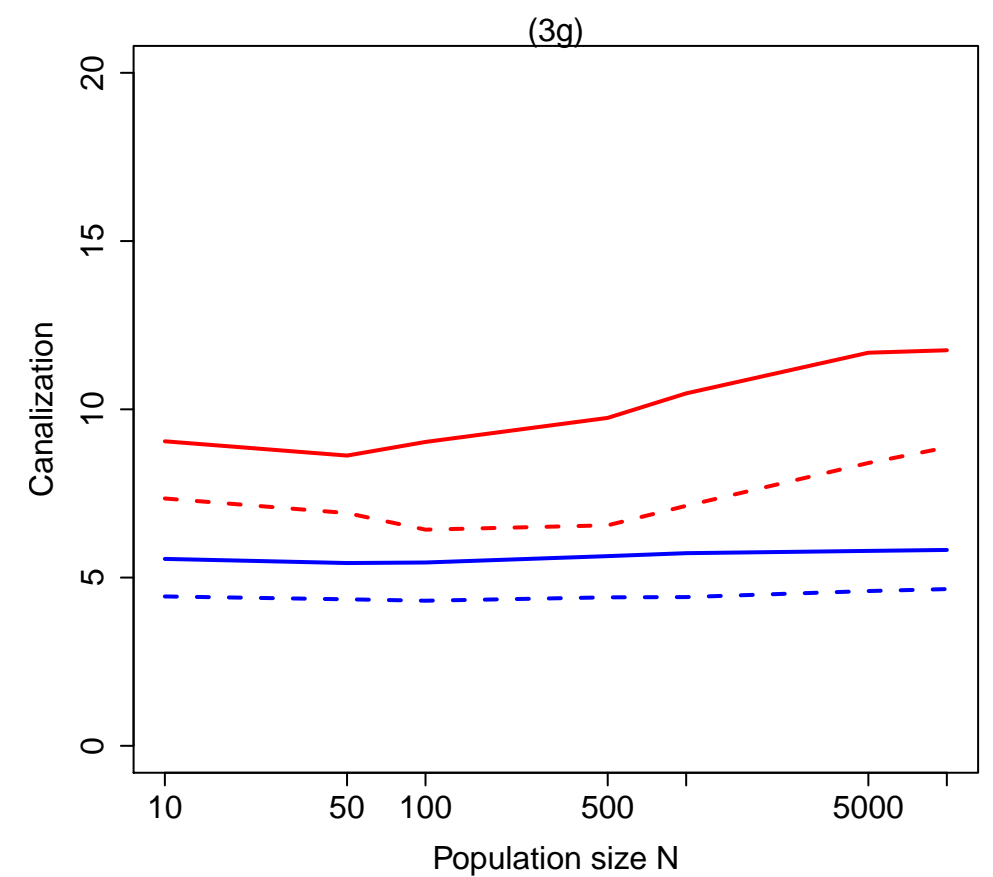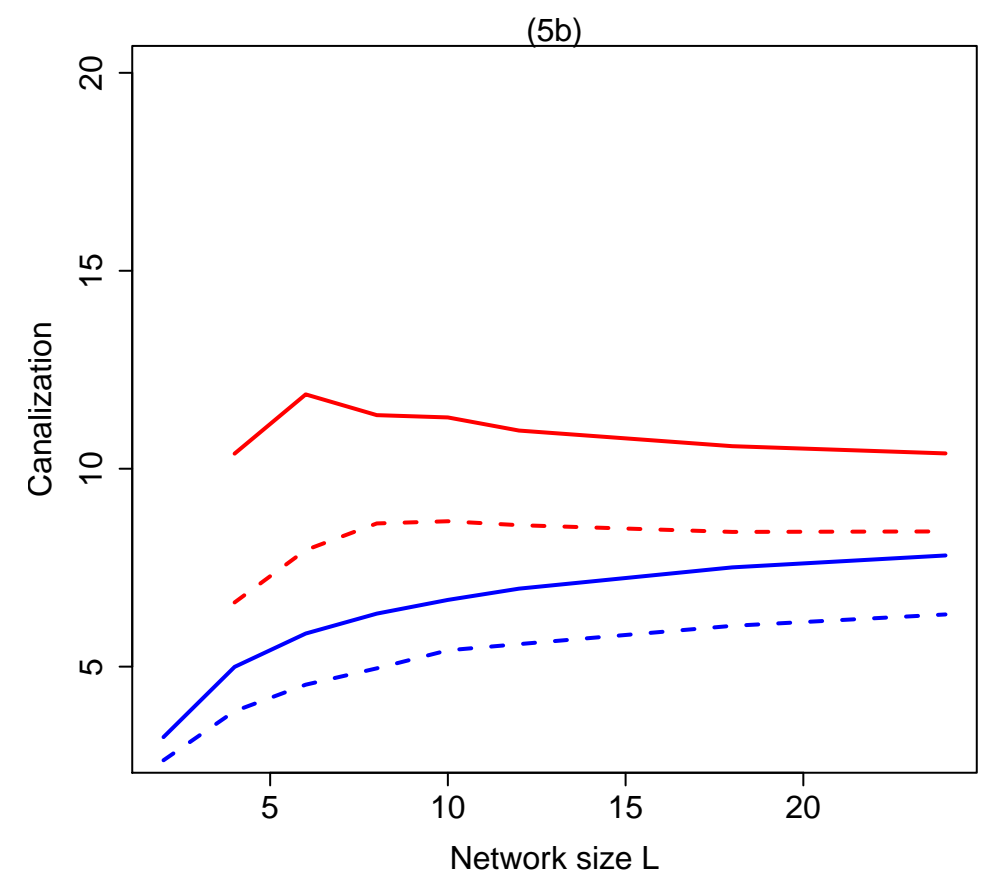

Supplement: Additional file 1 — Figures S2 and S1. Ploidy level. Figure S2. Effect of ploidy on the evolution of expression and canalization. Solid lines stand for diploid populations, dashed lines for haploid populations, and dotted lines for haploid populations with $\sigma _{m}^{\prime } = 2^{1/2} \sigma _{m}$σm′=21/2σm for the canalization tests (to compensate the increase in mutational effect that comes from the second unchanged haplotype in diploids). Subfigure indexes match figure numbers from the main text. (2a)(2b) Evolution of gene expression and canalization in networks in which genes are selected towards an intermediate optimum. (4a)(4b) Evolution of gene expression and canalization in networks in which genes are selected towards extreme expression. Figure S3. Effect of simulation parameters on canalization in haploid vs. diploid populations. Average and s.d. canalization scores at G=10,000 generations; two genes out of six are under direct selection pressure. Solid lines stand for diploid populations, dashed lines for haploid populations. Subfigure indexes match figure numbers from the main text. (3a) Network complexity c. (3b) Constitutive gene expression a. (3c) Mutation rate μ. (3d) Effect of a mutation σ m. (3e) Strength of stabilizing selection s. (3f) Fitness optimum θ. (5b) Population size N. (5b) Network size L. (ZIP 14 kb) [file 12862_2016_801_MOESM1_ESM.zip › supplementary_figure_3.pdf]

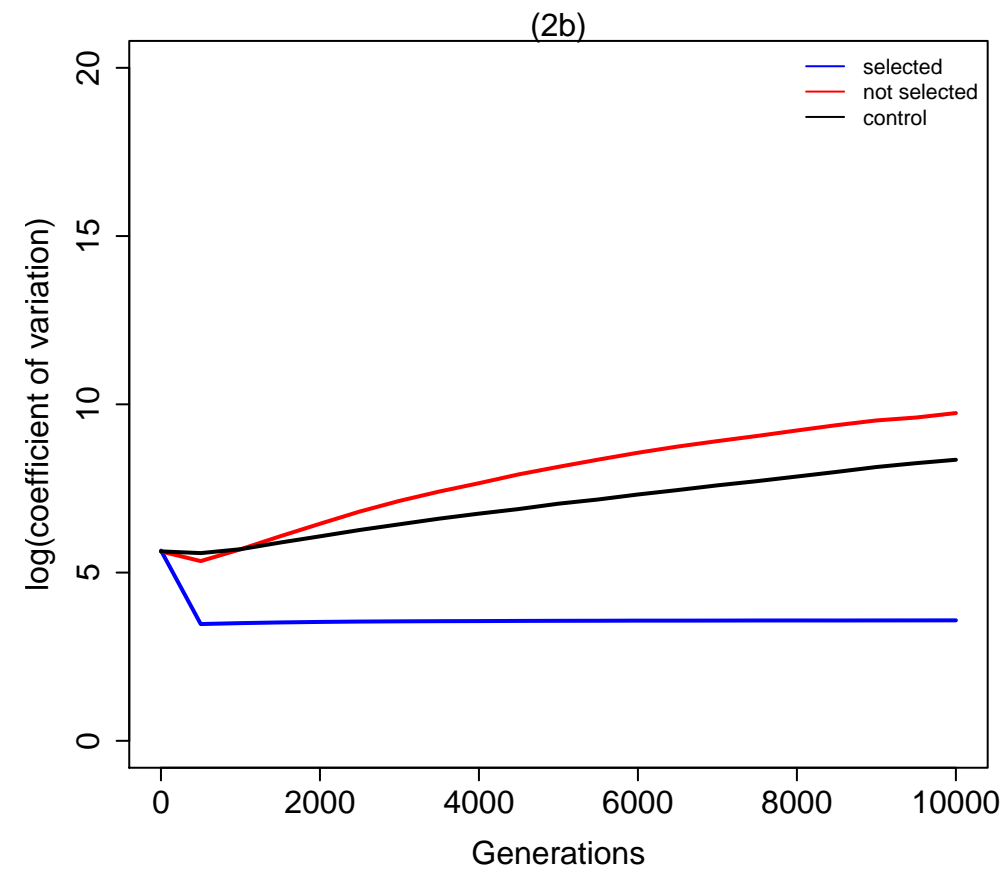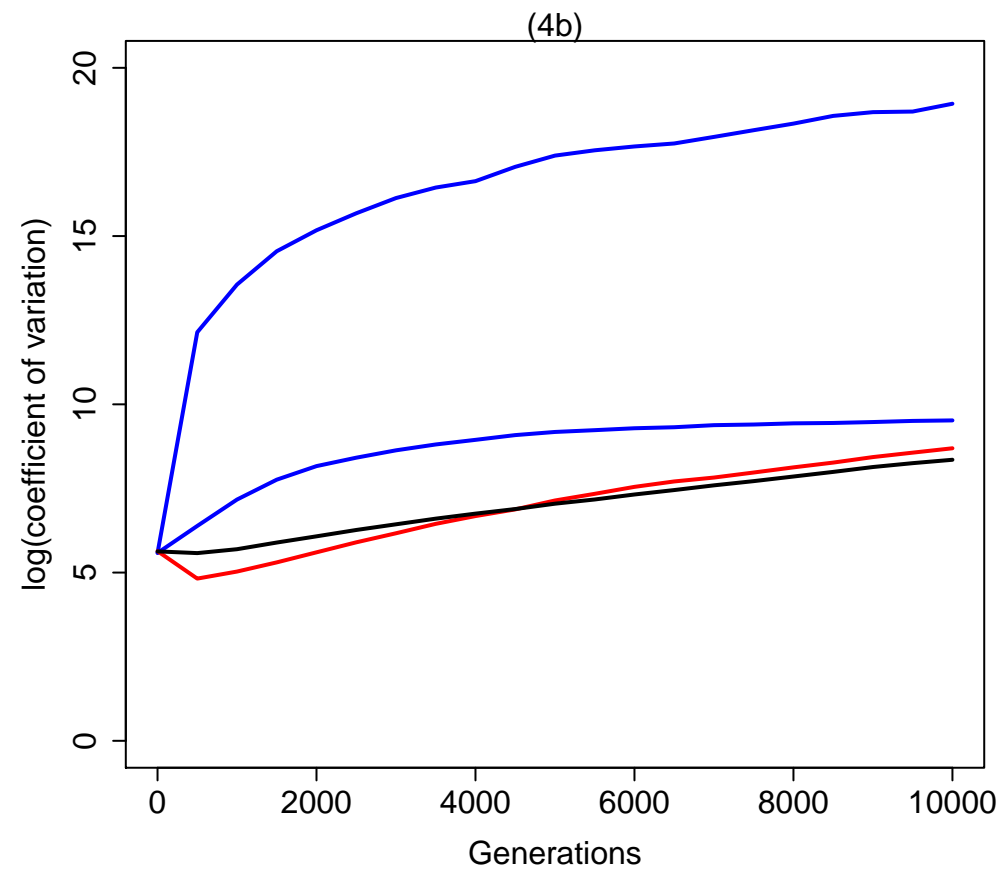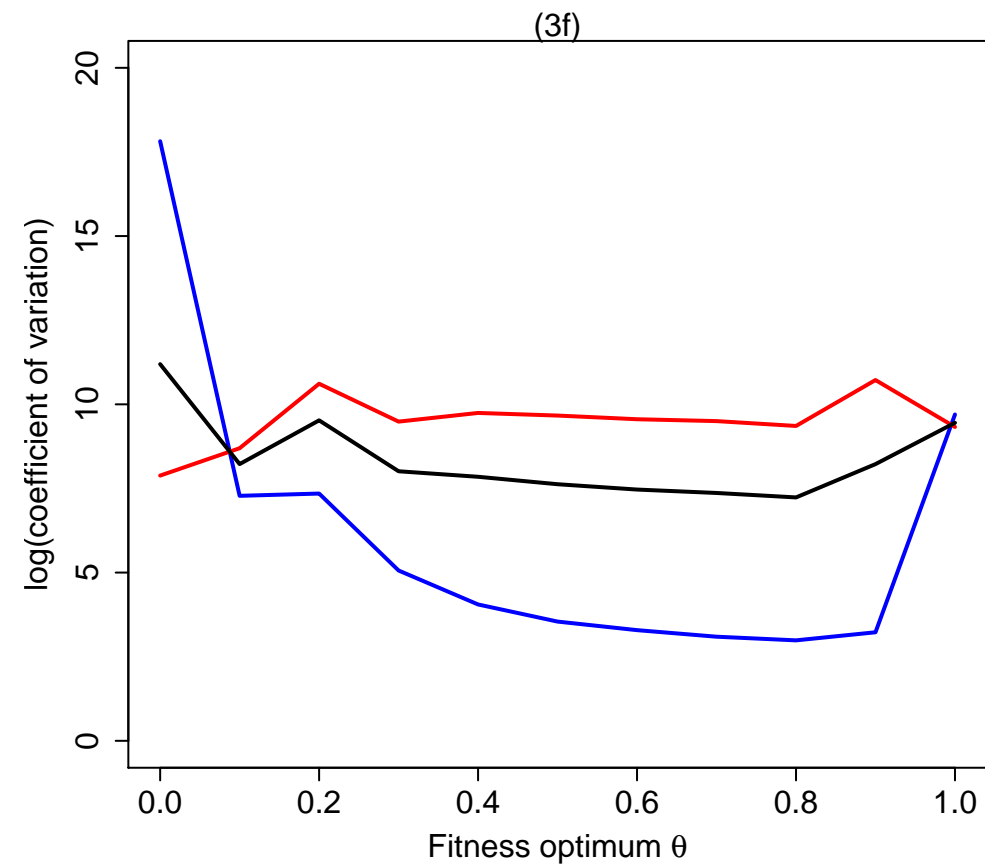

Supplement: Additional file 2 — Figure S3. Scale effect on canalization scores. Figure S4. Canalization measured as coefficient of variation. Evolution of canalization when measured relative to the phenotypic expression (i.e. in a similar way as a coefficient of variation: $C^{\prime }_{n_{i}} = \log \sqrt { ext {Var}(M_{n_{i}})}/S_{n_{i}}$Cni′=logVar(Mni)/Sni. In practice, we computed $\overline C^{\prime }_{n} \simeq \frac{1}{2}\overline {C}_{n} - \log \overline {S}_{n}$C¯n′≃12C¯n−logS¯n. Subfigure indexes match figure numbers from the main text, the Y axis represents $\overline C^{\prime }$C¯′ averaged over different categories of genes (selected, non-selected, and for subfigure (4b), selected for optima of 0 (top blue line) and 1 (bottom blue line)). (PDF 6 kb) [file 12862_2016_801_MOESM2_ESM.pdf]

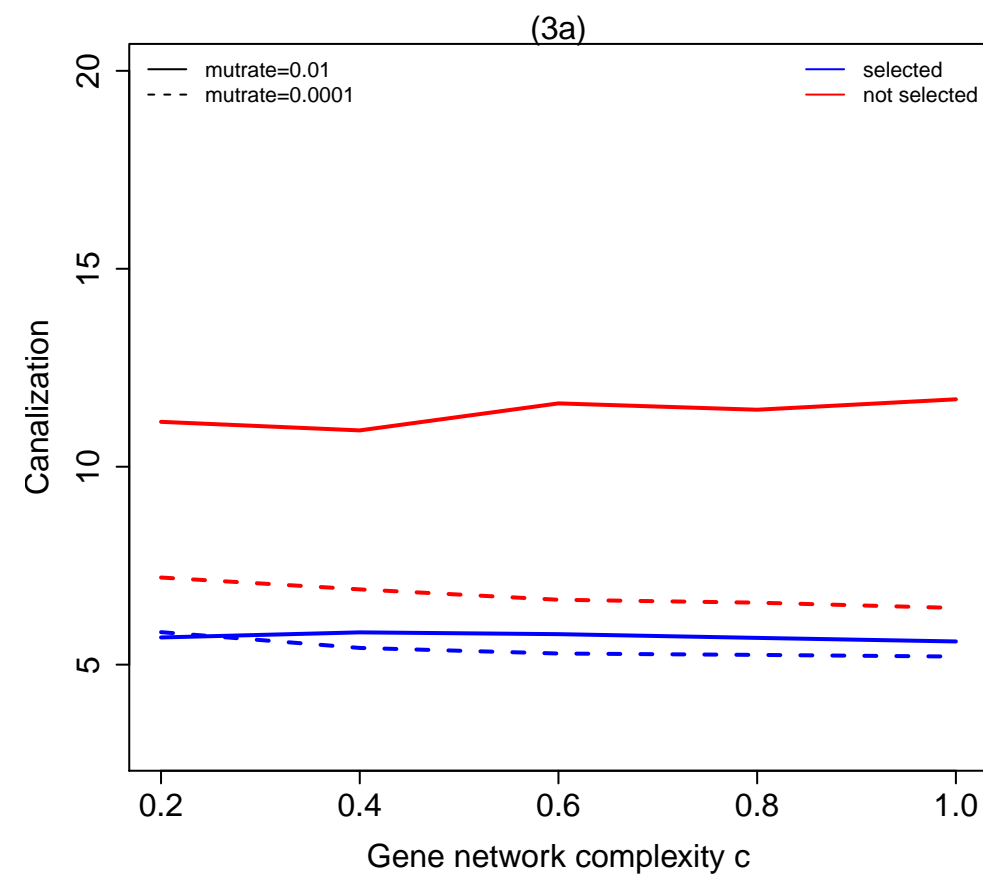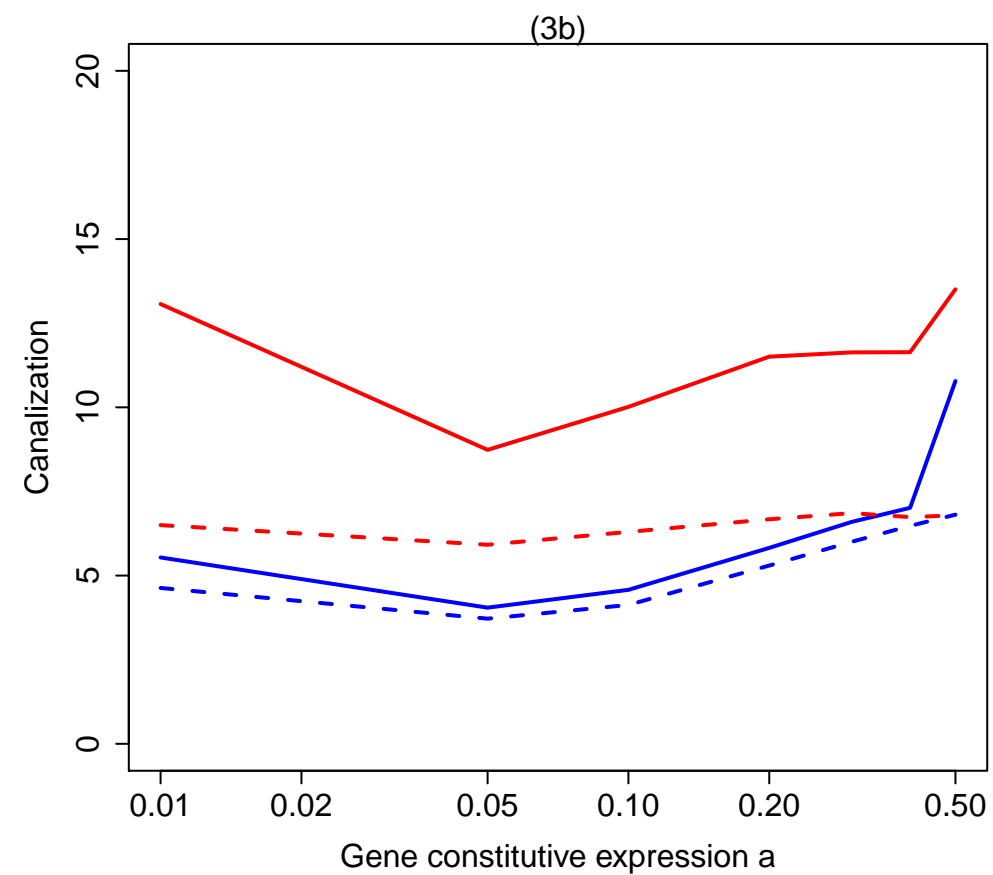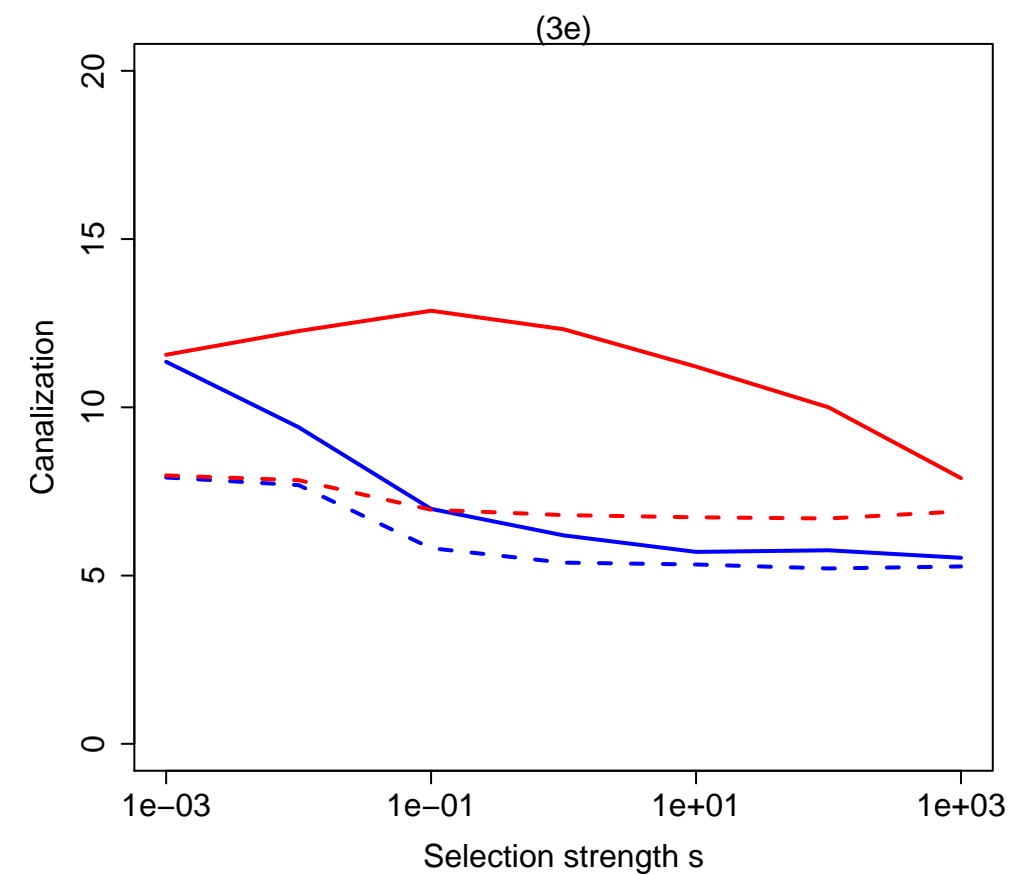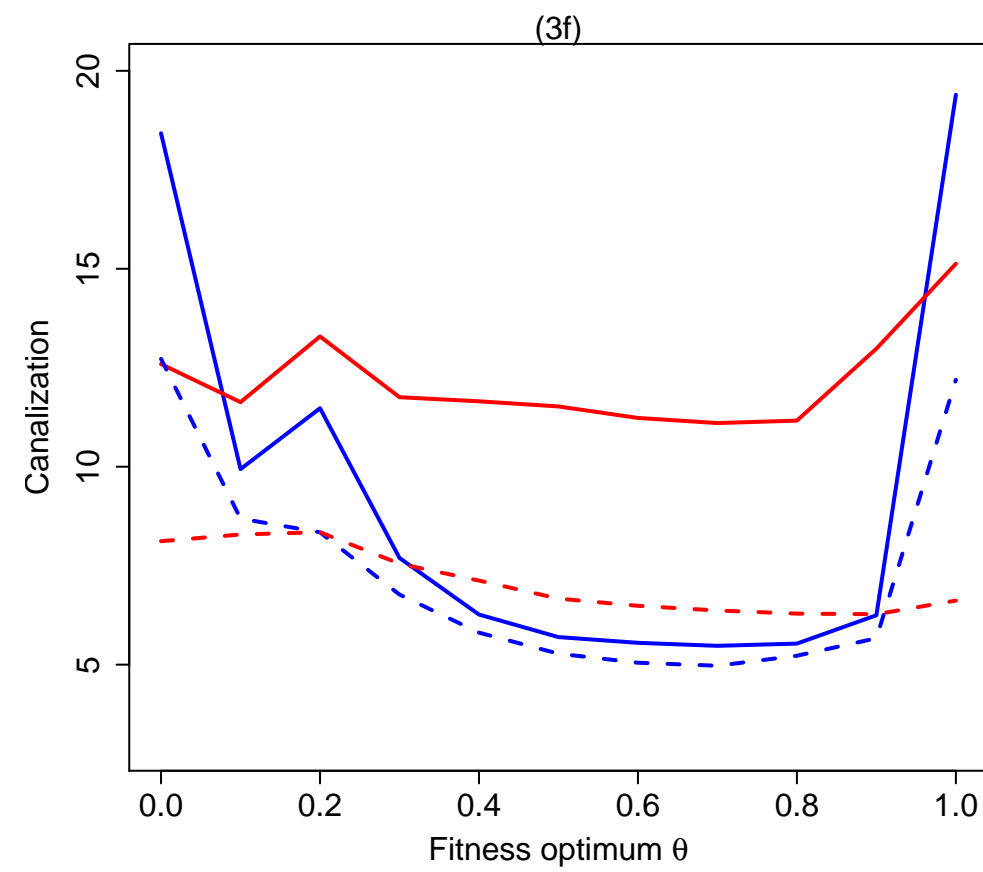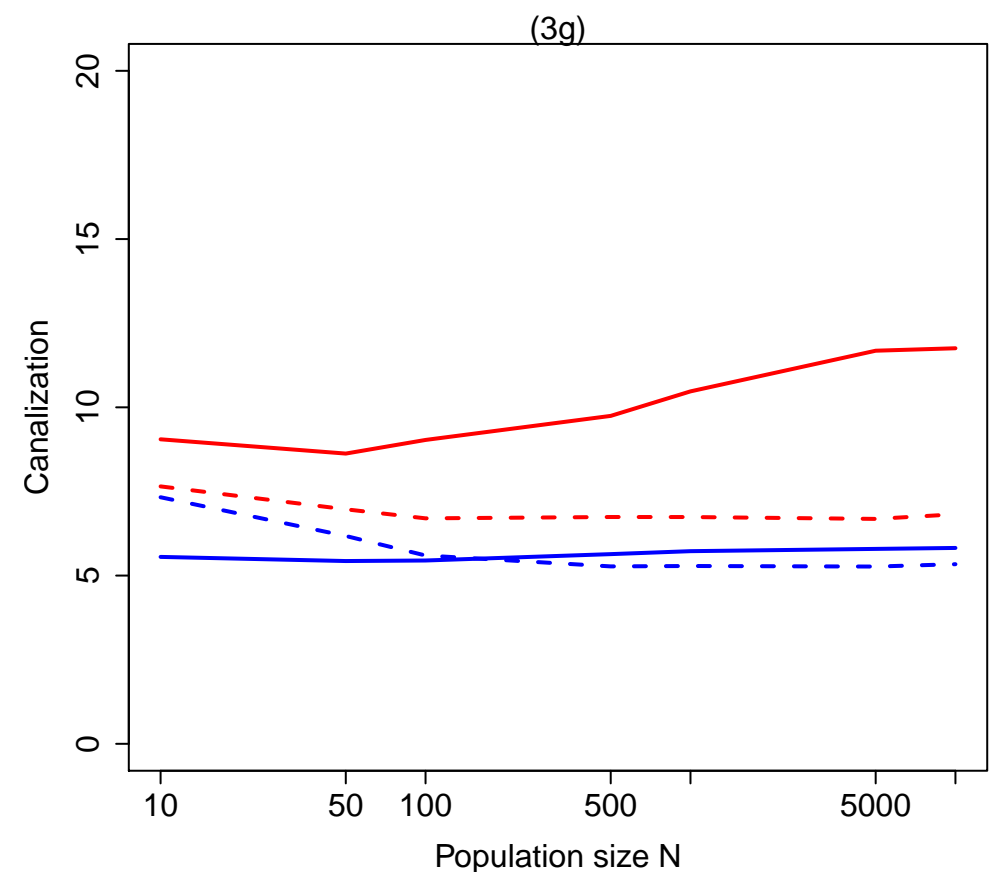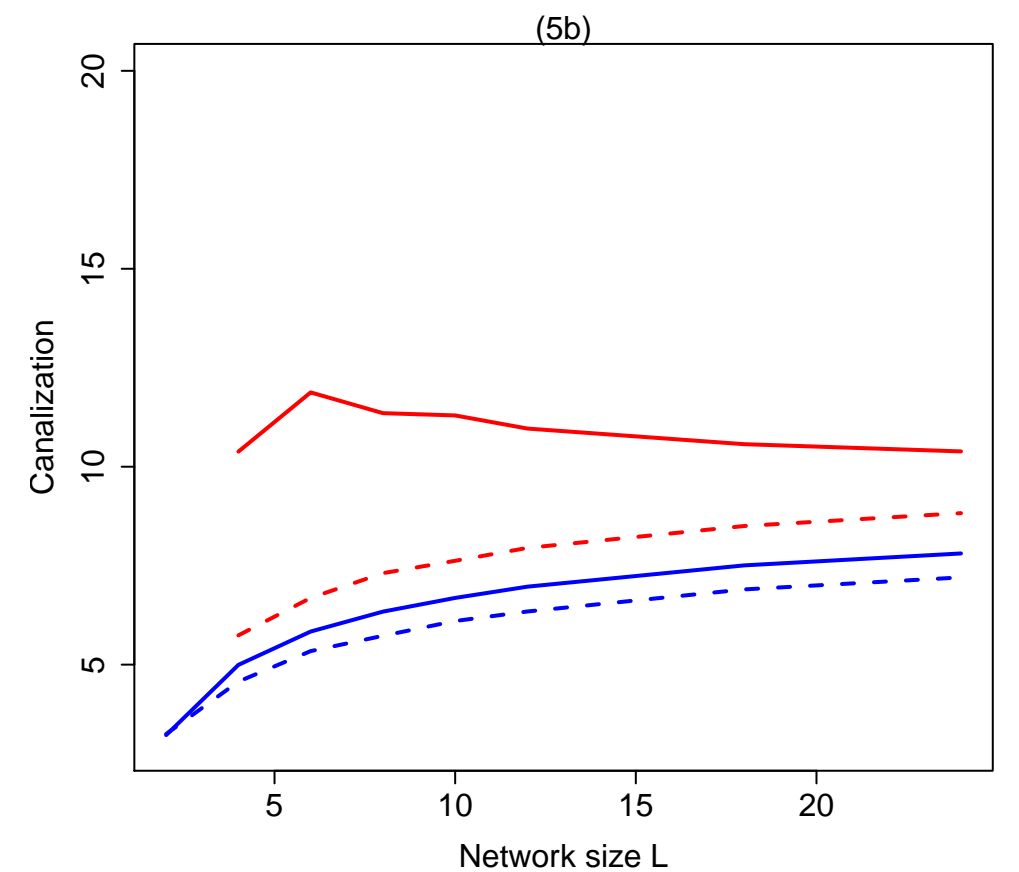

Supplement: Additional file 3 — Effect of mutation rate. Figure S4. Canalization still evolves at low mutation rates. Average canalization scores at G=10,000 generations and μ=0.0001 ; two genes out of six are under direct selection pressure. Subfigure indexes match figure numbers from the main text. (3a) Network complexity c. (3b) Constitutive gene expression a. (3e) Strength of stabilizing selection s. (3f) Fitness optimum θ. (3g) Population size N. (5b) Network size L. (PDF 6 kb) [file 12862_2016_801_MOESM3_ESM.pdf]
